# Mortality of lymphoma and myeloma in China, 2004–2017: an observational study

**DOI:** 10.1186/s13045-019-0706-9

**Published:** 2019-03-04

**Authors:** Weiping Liu, Jiangmei Liu, Yuqin Song, Xiaopei Wang, Maigeng Zhou, Lijun Wang, Jun Ma, Jun Zhu, Union for China Leukemia Investigators of the Chinese Society of Clinical Oncology, Union for China Lymphoma Investigators of the Chinese Society of Clinical Oncology

**Affiliations:** 1 Key Laboratory of Carcinogenesis and Translational Research (Ministry of Education), Department of Lymphoma, Peking University Cancer Hospital and Institute, Beijing, China; 2 National Center for Chronic and Noncommunicable Disease Control and Prevention, Chinese Center for Disease Control and Prevention, Beijing, China; 3 Harbin Institute of Hematology and Oncology, Harbin, China

**Keywords:** Lymphoma, Multiple myeloma, Mortality, Epidemiology

## Abstract

**Background:**

There is a dearth of accurate information about patterns of mortality of lymphoid neoplasms and temporal trends in China. In this nationwide mortality study, we aimed to assess the mortality of lymphoma and myeloma in 2017 and the changes in the trend from 2004 to 2016.

**Methods:**

Death certificate data obtained from the Chinese Center for Disease Control and Prevention’s disease surveillance points system (CDC-DSP) and population data from the National Bureau of Statistics of China were used in this study. We described the mortality of lymphoma and myeloma in 2017 by age group, sex, residence, and region and evaluated the temporal trend from 2004 to 2016 using joinpoint regression.

**Results:**

An estimated 52,000 deaths associated with lymphoma and myeloma occurred in 2017. The age-standardized mortality rate China (ASMRC) and age-standardized mortality rate worldwide (ASMRW) per 100,000 were 3.74 and 2.60, respectively. Males had higher ASMRC than females (4.54 vs. 2.91 per 100,000). The ASMRC in urban areas was significantly higher than that in rural areas (4.35 vs. 3.47 per 100,000). The age-specific mortality rate showed an upward trend with age and reached a maximum in the age group of over 85 years. In terms of regional variation, Eastern China had the highest mortality rate (3.43/100,000), followed by Central China (3.10/100,000) and Western China (3.02/100,000). The mortality rates of lymphoma and myeloma increased annually by 4.5% during the period 2004–2016, with a significant rapid upward trend in rural areas since 2007.

**Conclusions:**

The mortality of lymphoma and myeloma increased in China from 2004 to 2017. The rapid increase in disease burden in rural areas highlights new challenges for disease prevention and control strategies.

## Background

Lymphoid neoplasms, historically called lymphoid malignancies, comprise 5 major categories: Hodgkin lymphoma (HL), non-Hodgkin lymphoma (NHL), myeloma, acute lymphoid leukemia, and chronic lymphoid leukemia [1]. Lymphoid neoplasms are a common leading cause of death worldwide. A systematic analysis for the Global Burden of Disease Study 2016 demonstrated that the numbers of estimated all-age deaths due to lymphoid neoplasms were as follows: HL, 28,700; NHL, 239,600; multiple myeloma, 98,400; acute lymphoid leukemia, 50,900; and chronic lymphoid leukemia, 35,400 [2].

Different geographic distributions are observed between eastern and western countries. For example, in the USA, lymphoid neoplasms were the fourth most common cancer, with an expected 136,960 new cases in 2016 [3], and the mortality rate of lymphoid neoplasms per 100,000 population was 21.6 in 2014 (0.4 due to HL, 8.3 due to NHL, 3.9 due to multiple myeloma, 9.0 due to leukemia) [4]. A nationwide statistical analysis in Korea [5] showed a total of 6638 lymphoid malignancies in 2012, and the age-standardized incidence rates of all lymphoid malignancies increased from 6.9 to 9.9 per 100,000 persons during the period 1999–2012.

However, a comprehensive mortality description of these lymphoid neoplasms based on the national population, including differences in mortality according to sex, age group, residence, and region, has not yet been conducted in China. Ultimately, an understanding of mortality trends will help to direct future studies of disease control and prevention strategies. Therefore, this analysis sought to determine the mortality rates of lymphoma and myeloma in 2017, as defined by the World Health Organization classification [1], in mainland China. In addition, this study reports trends in mortality rates for lymphoma and myeloma from 2004 to 2016.

## Methods

### Data sources

Mortality data on patients with lymphoma and myeloma (International Classification of Diseases, 10 codes: C81–85, C88, C90, C96) between 2004 and 2017 were collected from the Chinese Center for Disease Control and Prevention’s disease surveillance points system (CDC-DSP). ICD-10 codes for each subtype of lymphoma and myeloma are listed in Table 1. The variables extracted were sex, age, year of diagnosis, residence (urban and rural areas), and region (Eastern China, Central China, and Western China).

**Table 1:** ICD-10 codes for each subtype of lymphoma and myeloma

Subtype	ICD-10
Hodgkin lymphoma	C81
Nodular lymphocyte predominant Hodgkin lymphoma	C81.0
Nodular sclerosis (classical) Hodgkin lymphoma	C81.1
Mixed cellularity (classical) Hodgkin lymphoma	C81.2
Lymphocyte-depleted (classical) Hodgkin lymphoma	C81.3
Lymphocyte-rich (classical) Hodgkin lymphoma	C81.4
Other (classical) Hodgkin lymphoma	C81.7
Hodgkin lymphoma, unspecified	C81.9
Follicular lymphoma	C82
Follicular lymphoma grade I	C82.0
Follicular lymphoma grade II	C82.1
Follicular lymphoma grade III, unspecified	C82.2
Follicular lymphoma grade IIIa	C82.3
Follicular lymphoma grade IIIb	C82.4
Diffuse follicle center lymphoma	C82.5
Cutaneous follicle center lymphoma	C82.6
Other types of follicular lymphoma	C82.7
Follicular lymphoma, unspecified	C82.9
Non-follicular lymphoma	C83
Small cell B cell lymphoma	C83.0
Mantle cell lymphoma	C83.1
Diffuse large B cell lymphoma	C83.3
Lymphoblastic (diffuse) lymphoma	C83.5
Burkitt lymphoma	C83.7
Other non-follicular lymphoma	C83.8
Non-follicular (diffuse) lymphoma, unspecified	C83.9
Mature T/NK cell lymphomas	C84
Mycosis fungoides	C84.0
Sézary disease	C84.1
Peripheral T cell lymphoma, not elsewhere classified	C84.4
Other mature T/NK cell lymphomas	C84.5
Anaplastic large cell lymphoma, ALK-positive	C84.6
Anaplastic large cell lymphoma, ALK-negative	C84.7
Cutaneous T cell lymphoma, unspecified	C84.8
Mature T/NK cell lymphoma, unspecified	C84.9
Other and unspecified types of non-Hodgkin lymphoma	C85
B cell lymphoma, unspecified	C85.1
Mediastinal (thymic) large B cell lymphoma	C85.2
Other specified types of non-Hodgkin lymphoma	C85.7
Non-Hodgkin lymphoma, unspecified	C85.9
Other specified types of T/NK cell lymphoma	C86
Extranodal NK/T cell lymphoma, nasal type	C86.0
Hepatosplenic T cell lymphoma	C86.1
Enteropathy-type (intestinal) T cell lymphoma	C86.2
Subcutaneous panniculitis-like T cell lymphoma	C86.3
Blastic NK cell lymphoma	C86.4
Angioimmunoblastic T cell lymphoma	C86.5
Primary cutaneous CD30-positive T cell proliferations	C86.6
Malignant immunoproliferative diseases	C88
Waldenström macroglobulinemia	C88.0
Other heavy chain diseases	C88.2
Immunoproliferative small intestinal disease	C88.3
Extranodal marginal zone B cell lymphoma of mucosa-associated lymphoid tissue [MALT lymphoma]	C88.4
Other malignant immunoproliferative diseases	C88.7
Malignant immunoproliferative disease, unspecified	C88.9
Multiple myeloma and malignant plasma cell neoplasms	C90
Multiple myeloma	C90.0
Plasma cell leukemia	C90.1
Extramedullary plasmacytoma	C90.2
Solitary plasmacytoma	C90.3
Other and unspecified malignant neoplasms of lymphoid, hematopoietic, and related tissue	C96
Multifocal and multisystemic (disseminated) Langerhans cell histiocytosis [Letterer-Siwe disease]	C96.0
Malignant mast cell tumor	C96.2
Sarcoma of dendritic cells (accessory cells)	C96.4
Multifocal and unisystemic Langerhans cell histiocytosis	C96.5
Unifocal Langerhans cell histiocytosis	C96.6
Other specified malignant neoplasms of lymphoid, hematopoietic, and related tissue	C96.7
Histiocytic sarcoma	C96.8
Malignant neoplasm of lymphoid, hematopoietic, and related tissue, unspecified	C96.9

A total of 161 surveillance points across 31 provinces were included in this population-based death registration system from 2004 to 2012, providing a population coverage of approximately 73 million people (approximately 6% of the total Chinese population). This system consists of 605 surveillance points and covers a population of 323.8 million (24.3% of the total population of the country) since 2013. Good national and regional representativeness of the system have been determined in previous studies [6–8], and detailed information about this database has been published elsewhere [9]. Multiple strategies for addressing variations in data quality are routinely implemented, including regular checking, supervision, feedback, and verification. In addition, two underreporting surveys that aimed to evaluate the data completeness of the CDC-DSP system were conducted during 2006–2008 and 2009–2011 [10, 11]. The mortality rates were adjusted by underreporting rates in each corresponding year according to the following formula: estimated mortality rates = reported mortality rates/(1—underreporting rates).

National age-specific population data were obtained from the National Bureau of Statistics of China (http://data.stats.gov.cn). At the end of 2017, there were 1,390,080,000 persons, including 711,370,000 males and 678,710,000 females, in mainland China, of which 813,470,000 resided in urban areas and 576,610,000 resided in rural areas. The products of the age-specific mortality rates and corresponding population in each stratum were added up to calculate the estimated deaths due to lymphoma and myeloma in 2017. The whole-population data were divided by age group (0–1, 1–84 in 5-year intervals and 85+ years). The 2010 census population of China and Segi’s population were used to determine the age-standardized mortality rate China (ASMRC) and the age-standardized mortality rate world (ASMRW), respectively.

### Statistical analysis

The numbers of deaths and mortality rates of lymphoma and myeloma in 2017 were estimated using the CDC-DSP database. Temporal trends in mortality rates from 2004 to 2017 were examined by IBM SPSS Statistics for Windows (version 21.0; IBM Corp.) and fitting joinpoint models (version 4.6.0.0; National Cancer Institute). The trends were expressed as annual percentage changes (APCs), and *Z* tests were used to assess whether the APCs were significantly different from zero. In describing trends, the terms “increase” and “decrease” were used when the slope of the trend was statistically significant; otherwise, the term “stable” was used. Statistical significance was assessed at the 0.05 level, and all hypothesis tests were two-sided.

## Results

### Expected deaths and mortality rates of lymphoma and myeloma in 2017

An estimated 52,000 deaths associated with lymphoma and myeloma occurred, and the crude mortality rate was 3.83 per 100,000 in 2017. The ASMRC and ASMRW per 100,000 were 3.74 and 2.60, respectively (Table 2). Males had higher mortality rates than females. The mortality rates in urban areas were significantly higher than those in rural areas.

**Table 2:** Mortality rates of lymphoma and myeloma in 2017

	Sex	Deaths (× 10^3^)	Crude rate (1/10^5^)	ASMRC (1/10^5^)	ASMRW (1/10^5^)
All	Both	52	3.83	3.74	2.60
	Male	32	4.67	4.54	3.30
	Female	20	2.97	2.91	1.93
Urban	Both	29	4.43	4.35	2.97
	Male	18	5.28	4.53	3.73
	Female	11	3.57	2.91	2.25
Rural	Both	23	3.52	3.74	2.42
	Male	14	4.35	4.53	3.09
	Female	9	2.66	2.91	1.77

*ASMRC*, age-standardized mortality rate adjusted by the Chinese standard population; *ASMRW*, age-standardized mortality rate adjusted by the world standard population

### Age-specific mortality rates of lymphoma and myeloma stratified by residence and sex

**Fig. 1:**
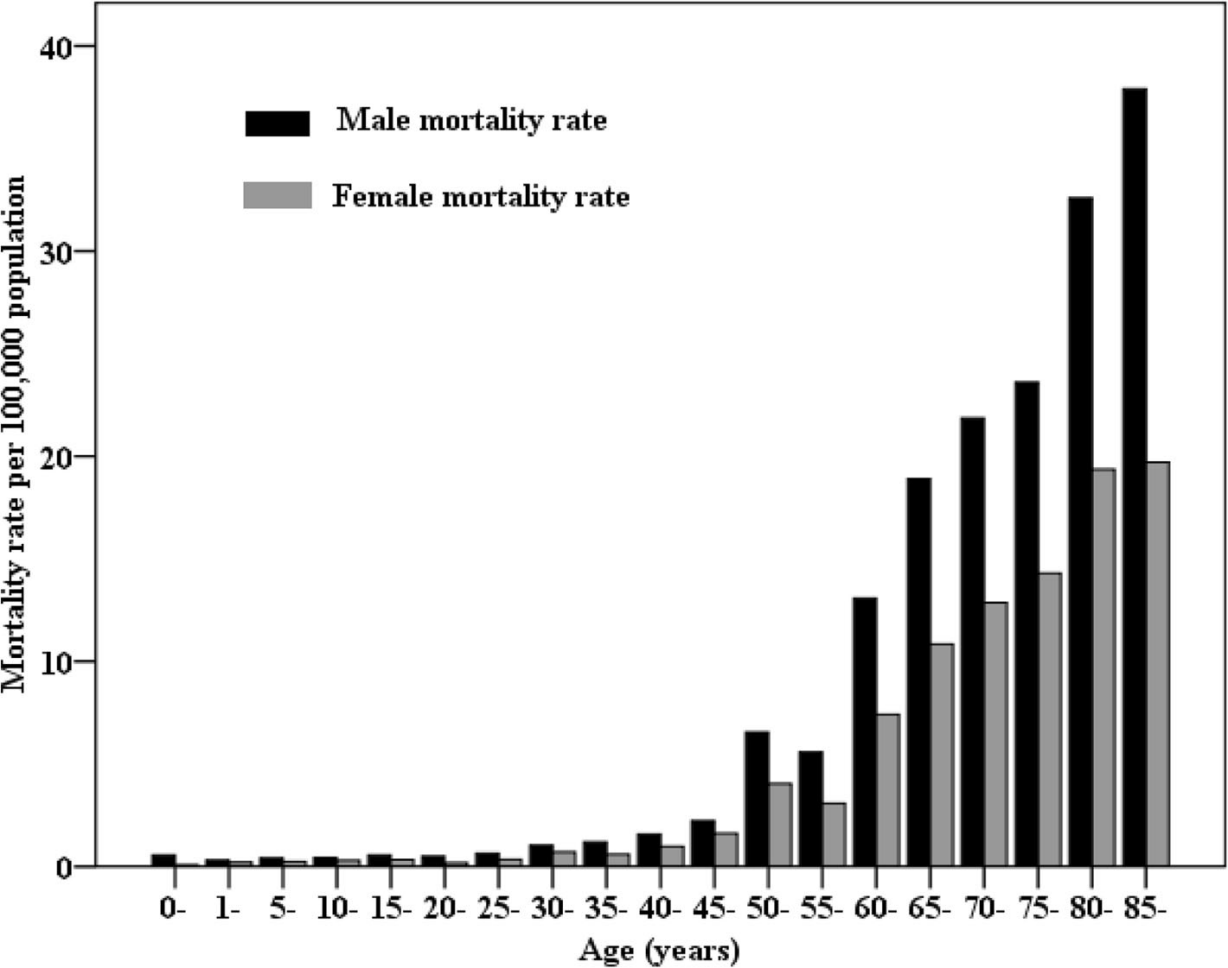
Age-specific mortality of lymphoma and myeloma by sex in China, 2017

Higher mortality rates were observed in older individuals. Age-specific mortality rates showed an upward trend with age and reached a maximum in the age group of over 85 years (Table 3). Males had higher mortality rates than females in all age groups (Fig. 1). In terms of residence variation, age-specific mortality rates in urban areas were higher than those in rural areas after the age of 55 years. Males over 85 years of age in urban areas had the highest mortality rates.

**Table 3:** Age-specific mortality rates of lymphoma and myeloma, stratified by residence, 2017 (1/10^5^)

Age group	All areas			Urban areas			Rural areas		
	Both	Male	Female	Both	Male	Female	Both	Male	Female
0–1	0.34	0.56	0.08	0.00	0.00	0.00	0.46	0.74	0.11
1–4	0.26	0.30	0.20	0.17	0.19	0.15	0.29	0.3	0.22
5–9	0.33	0.41	0.24	0.32	0.40	0.22	0.34	0.42	0.25
10–14	0.37	0.44	0.30	0.37	0.51	0.20	0.38	0.41	0.34
15–19	0.45	0.55	0.33	0.40	0.53	0.26	0.47	0.57	0.37
20–24	0.34	0.49	0.18	0.21	0.26	0.16	0.41	0.62	0.19
25–29	0.49	0.65	0.34	0.46	0.60	0.32	0.51	0.67	0.35
30–34	0.86	1.02	0.70	0.86	1.12	0.60	0.86	0.96	0.76
35–39	0.89	1.17	0.59	0.79	0.93	0.65	0.95	1.32	0.56
40–44	1.26	1.55	0.96	1.13	1.13	1.13	1.33	1.77	0.87
45–49	1.90	2.22	1.59	1.80	1.91	1.70	1.96	2.38	1.54
50–54	5.31	6.53	4.03	5.29	6.82	3.66	5.32	6.38	4.22
55–59	4.33	5.57	3.07	4.67	6.13	3.20	4.14	5.27	3.00
60–64	10.27	13.06	7.41	12.24	15.30	9.12	9.35	12.01	6.61
65–69	14.85	18.90	10.83	17.31	21.82	12.86	13.73	17.57	9.91
70–74	17.28	21.86	12.85	20.07	24.42	16.06	15.86	20.61	11.14
75–79	18.67	23.61	14.29	23.94	31.02	17.77	15.89	19.75	12.43
80–84	25.23	32.59	19.35	37.29	44.39	31.39	19.45	26.73	13.76
85+	26.69	37.90	19.70	43.76	60.33	32.72	18.64	26.66	13.81

### Age-specific mortality rates of lymphoma and myeloma stratified by region

In total, Eastern China had the highest mortality rate (3.43/100,000), followed by Central China (3.10/100,000) and Western China (3.02/100,000). Upward trends with age were observed in all regions (Table 4). Western China had higher mortality rates in the age group of 0–1 years than Eastern China and Central China. Males over 85 years of age in Eastern China had the highest mortality rates.

**Table 4:** Age-specific mortality rates of lymphoma and myeloma by region, 2017 (1/10^5^)

Age group	Eastern China			Central China			Western China		
	Both	Male	Female	Both	Male	Female	Both	Male	Female
0–1	0.30	0.56	0.00	0.31	0.57	0.00	0.43	0.54	0.31
1–4	0.20	0.23	0.17	0.25	0.36	0.11	0.35	0.33	0.38
5–9	0.32	0.34	0.29	0.27	0.33	0.20	0.43	0.63	0.23
10–14	0.31	0.28	0.34	0.44	0.60	0.24	0.38	0.45	0.31
15–19	0.32	0.36	0.28	0.51	0.67	0.33	0.53	0.65	0.40
20–24	0.27	0.35	0.17	0.34	0.47	0.21	0.45	0.74	0.16
25–29	0.49	0.72	0.26	0.52	0.67	0.38	0.46	0.48	0.44
30–34	0.84	0.94	0.73	0.79	0.93	0.65	1.00	1.27	0.72
35–39	0.97	1.28	0.65	0.97	1.35	0.59	0.68	0.84	0.52
40–44	1.27	1.35	1.19	1.06	1.39	0.73	1.52	2.06	0.95
45–49	1.81	1.99	1.63	1.77	2.05	1.50	2.23	2.77	1.68
50–54	4.64	5.79	3.43	5.69	6.98	4.36	6.08	7.34	4.74
55–59	4.87	6.13	3.59	4.16	5.55	2.76	3.61	4.62	2.60
60–64	11.56	14.54	8.54	9.36	12.14	6.51	9.33	11.84	6.72
65–69	16.78	21.57	12.12	14.72	18.99	10.44	11.87	14.49	9.23
70–74	18.94	24.18	14.00	15.99	21.21	10.81	16.24	18.95	13.59
75–79	20.16	25.38	15.69	18.15	22.14	14.47	16.53	22.42	11.21
80–84	28.52	36.38	22.69	23.92	30.54	18.29	19.95	27.91	13.17
85+	30.27	43.30	22.53	24.14	34.85	17.31	22.45	31.00	16.74

### Trends in mortality of lymphoma and myeloma

**Fig. 2:**
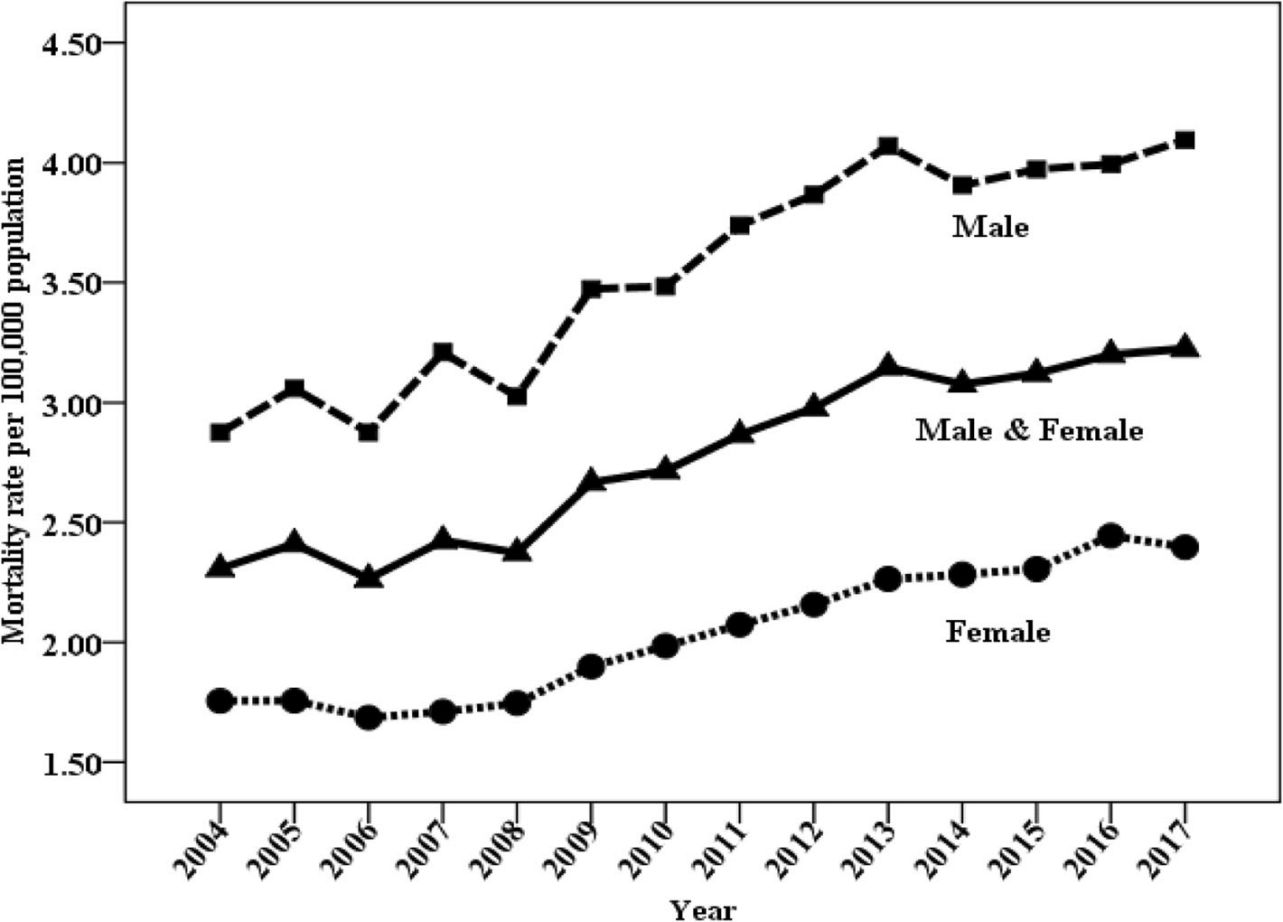
Trends in mortality of lymphoma and myeloma (age-standardized to the Chinese standard population) by sex: China, 2004 to 2017

**Fig. 3:**
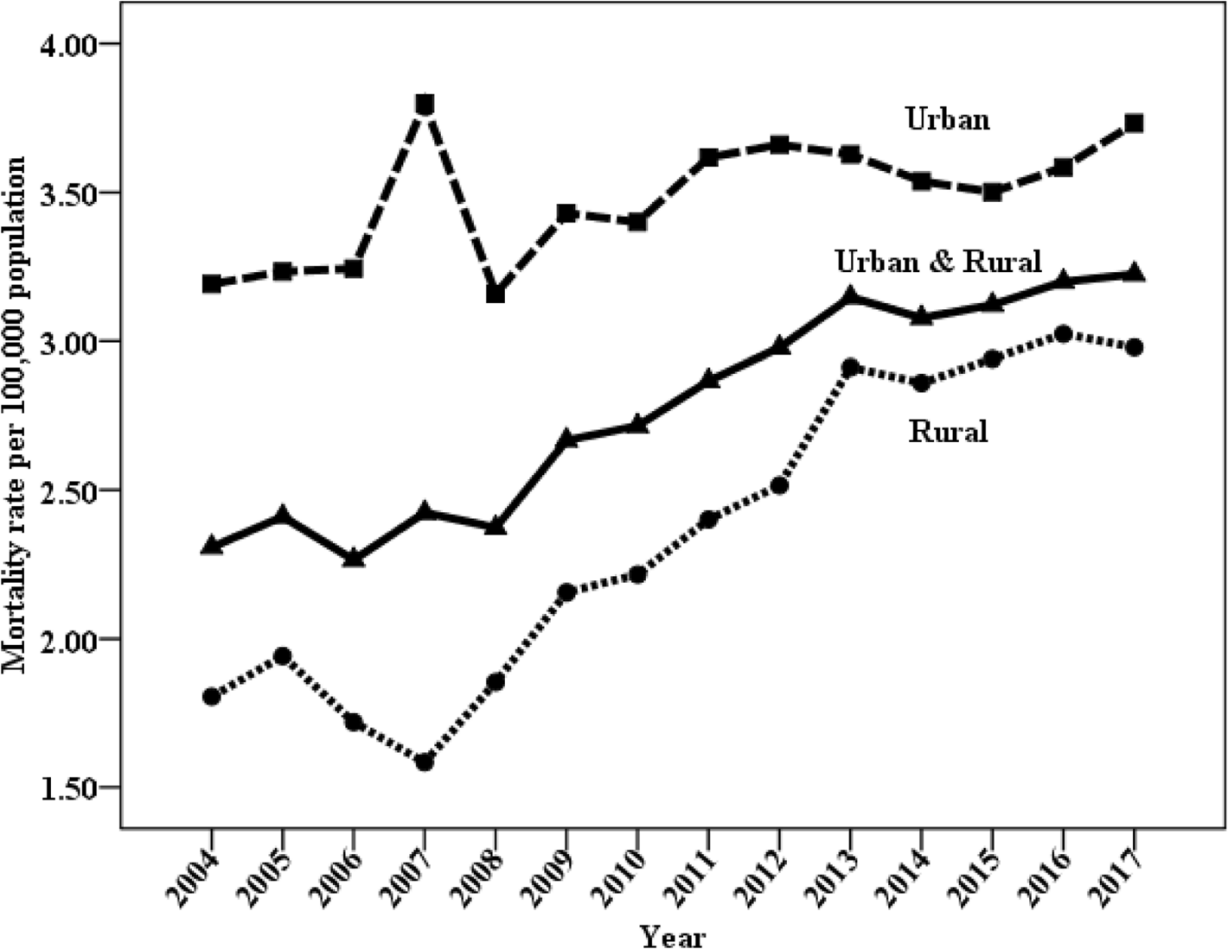
Trends in mortality of lymphoma and myeloma (age-standardized to the Chinese standard population) by residence: China, 2004 to 2017

The temporal trends in mortality of lymphoma and myeloma by sex and residence over the study period (2004–2016) are illustrated in Figs. 2 and 3 and Table 5. The age-standardized mortality rates increased for both males and females (Fig. 2). Significant upward trends were observed in both urban and rural areas. Notably, the mortality rates in rural areas increased rapidly starting in 2007 (Fig. 3).

**Table 5:** Trends in mortality of lymphoma and myeloma (age-standardized to the Chinese standard population) by sex and residence in China, 2004 to 2016

Sex		Trend 1#	Trend 2#			Trend 3#	
		Year	APC	Year	APC	Year	APC
All	Both	2004–2016	4.5*				
	Male	2004–2016	4.4*				
	Female	2004–2006	− 1.2	2006–2016	5.5*		
Urban	Both	2004–2016	2.3*				
	Male	2004–2016	1.9*				
	Female	2004–2016	2.9*				
Rural	Both	2004–2016	6.8*				
	Male	2004–2016	6.7*				
	Female	2004–2007	− 2.3	2007–2016	9.1*		

*The annual percentage change is significantly different from zero

^#^Each change in magnitude and/or direction of trend is listed separately with the years for which that trend was constant. Therefore, if only one trend is listed for 2004 through 2017, that trend was constant during the entire time period

## Discussion

According to the statistics of GLOBOCAN 2018, produced by the International Agency for Research on Cancer, lymphoid neoplasms accounted for 7.2% of the 9.6 million cancer deaths worldwide in 2018, including 0.3% of deaths due to HL, 2.6% due to NHL, 1.1% due to multiple myeloma, and 3.2% due to leukemia [12]. Compared with the statistics of GLOBOCAN 2012 [13], both new cases and deaths associated with lymphoid neoplasms increased. For example, the estimated number of new NHL cases increased from 385,700 in 2012 to 509,590 in 2018, while deaths increased from 199,700 in 2012 to 248,724 in 2018. A report [14] from the National Central Cancer Registry of China (NCCRC) estimated that lymphoid neoplasms accounted for 2.1% (88,200 new cases) of all new cancer cases and 1.9% (52,100 deaths) of all cancer deaths in 2015. In our study, the estimated number of deaths associated with lymphoma and myeloma reached 52,000 in 2017 due to the growth and aging of the population, meaning that approximately 7.5% of deaths associated with lymphoid neoplasms worldwide occurred in China.

Combined factors such as age and sex may have an important impact on variations in mortality rate. As with many other cancers, the likelihood of an individual being diagnosed with lymphoma increases markedly with age [15], and adolescent and young adult patients have better survival rates [16], all of which result in higher mortality rates in older individuals. Moreover, males have higher mortality rates than females, with some explained risk factors, such as smoking and infections [17]. A study from the USA [3] demonstrated that the overall incidence rates of lymphoid neoplasms were 51% higher in males than in females, and black men had the lowest survival across lymphoid neoplasm subtypes. Similarly, a study from China [18] indicated that males had higher age-standardized incidence rates (4.89 vs. 3.49 per 100,000) and mortality rates (2.84 vs. 1.75 per 100,000) of lymphoid neoplasms using the world standard population. Consistent with previous reports, higher mortality rates of lymphoma and myeloma were observed in males and older individuals in our study. In addition, geographical differences in mortality rates were observed, especially a higher mortality rate in the 0–1 year age group, which was observed in Western China due to an imbalance in socioeconomic development. Based on these disparities in disease burden in different specific populations, different strategies for disease prevention and control should be employed.

Compared with western countries, China had a lower disease burden of lymphoid neoplasms. In the European standard population, the age-standardized incidence and mortality rates of lymphoid neoplasms per 100,000 population in 2018 were 30.5 and 12.6, respectively [19]. In China, lower age-standardized incidence rates (4.18 per 100,000) and mortality rates (2.28 per 100,000) using the world standard population were estimated in 2015 [14]. However, the disease burden of lymphoid neoplasms has been rising in China over the last decade. In our study, the mortality rates of lymphoma and myeloma increased annually by 4.5% during the period of 2004–2016, with increasing trends in both sexes across all areas. These upward trends may be explained partly by improvements in diagnostic procedures and changes in the classification of lymphoid neoplasms [1], but much of these trends may reflect an increase in deaths due to poor survival [20]. The prognosis of patients with lymphoid neoplasms was poor in China, with a 5-year relative survival rate of 38.3%, while it improved markedly, with a survival rate of 70% or higher, in European countries during the same period [21]. Similarly, the 5-year survival of adults with lymphoid neoplasms and children with lymphoma improved from 61.2% and 88.5% in 2000–2004 to 68.1% and 94.3% in 2010–2014 in the USA, respectively [21]. A study from the NCCRC [22] demonstrated that the age-standardized 5-year relative survival rates of lymphoid neoplasms increased by less than 5% in China (changing from 32.6% during 2003–2005 to 37.2% during 2012–2015). Therefore, further studies focused on critical factors affecting mortality, such as lifestyle and treatment models among different countries, should be executed.

Several factors contributed to the urban-rural discordance of mortality rates of lymphoma and myeloma in China. Urban areas had higher incidence rates of lymphoid neoplasms than rural areas in China (4.7 per 100,000 vs. 3.4 per 100,000) [18]. Poor availability of medical services [23] and insufficient protection by healthcare insurance [24] also played important roles. There were substantial social and health inequalities between urban and rural areas in China [25]. Compared to urban residents, both rich and poor village residents were less likely to use outpatient services, with odds ratios (ORs) of 0.728 and 0.778, and were less likely to use inpatient health care, with ORs of 0.609 and 0.752, respectively [26]. Patients in rural areas had lower survival rates due to difficulty in diagnosis and delayed or reduced access to appropriate therapies, regardless of health service improvements [27]. Furthermore, rural migrants (approximately 220 million) living and working in urban areas but without gaining formal urban medical insurance created additional challenges for the provision of optimum health care [28]. Remarkable progress has been made by the Chinese government toward upgrading the health-care system. The insurance coverage increased from 29.7% in 2003 to 95.7% in 2011, while the average share of inpatient costs reimbursed from insurance increased from 14.4 to 46.9 [29]. However, there were still large challenges on the way to eliminating the disparities between urban and rural areas due to the vast population, large geographical span, and diverse cultures and socioeconomic groups [30]. These findings highlighted the need to increase medical service accessibility and affordability to modern diagnosis and treatment, particularly for rural areas of China.

Of note was an increase in mortality rates of lymphoma and myeloma, especially a rapid increase since 2007 in rural areas. Between 2004 and 2016, the mortality rates increased by 4.5% for all areas, 2.3% for urban areas, and 6.8% for rural areas, respectively. Similar to our findings, a subnational analysis for the Global Burden of Disease Study 2013 showed that the age-standardized mortality rates increased with changes of 1.3% for NHL and 7.3% for multiple myeloma from 1990 to 2013 in China [6]. These results could mainly be attributable to the increase of incidence. From 2006 to 2016, the age-standardized years lived with disability rates in China increased by 4.9% for HL, 59.4% for NHL, and 48.6% for multiple myeloma, respectively [2]. Moreover, the increase in mortality rates during the period of 2013–2017 may be interpreted partly by improvements in the CDC-DSP system. Since 2013, the number of surveillance points increased from 161 to 605, and the surveillance population increased from 6 to 24% of the Chinese population, which strengthened both nationally and regionally (i.e., eastern, central, and western; urban and rural), representative of the CDC-DSP system since 2013 [9].

The interpretation of our study has several limitations. First, the accuracy of the cause of death was not assessed when the mortality counts were extracted from the CDC-DSP database. Because of the difficulty in diagnosing lymphoid neoplasms, the rates in the present study may be underestimated, especially in rural areas. Second, the changes in CDC-DSP surveillance points and population coverage since 2013 should also be considered in a cautious interpretation of the mortality trends. Third, there was still room for improvement in the quality of the CDC-DSP data; such an improvement would modify our estimates but not bias our main findings.

## Conclusions

This is the first study to present spatiotemporal variation in the mortality of lymphoma and myeloma in China using national data routinely collected from death certificates. Because the CDC-DSP system had a good national representativeness, with a coverage of 24.3% of the total Chinese population, our data determined the pattern of mortality rates of lymphoma and myeloma in China. Higher mortality rates were observed in males and in older individuals residing in urban areas. A rapid increase in mortality rates in rural areas was noted. The study results provided information on the disease burden of lymphoma and myeloma and allowed the monitoring of temporal trends in mortality in the Chinese population, which will be useful for policy-making with respect to the development of management strategies under circumstances of limited prevention measures.

## Abbreviations

APC: Annual percentage change
ASMRC: Age-standardized mortality rate China
ASMRW: Age-standardized mortality rate world
CDC-DSP: Center for Disease Control and Prevention’s Disease Surveillance Points System
HL: Hodgkin lymphoma
NCCRC: National Central Cancer Registry of China
NHL: Non-Hodgkin lymphoma
